# Mobile Acceptance and Commitment Therapy in Bipolar Disorder: Microrandomized Trial

**DOI:** 10.2196/43164

**Published:** 2023-04-20

**Authors:** Amy Cochran, Jacob M Maronge, Amanda Victory, Sydney Hoel, Melvin G McInnis, Emily BK Thomas

**Affiliations:** 1 Department of Population Health Sciences University of Wisconsin Madison Madison, WI United States; 2 Department of Mathematics University of Wisconsin Madison, WI United States; 3 Department of Biostatistics Monroe Dunaway Anderson Cancer Center Houston, TX United States; 4 Department of Psychiatry University of Michigan Ann Arbor, MI United States; 5 Department of Medicine University of Wisconsin Madison Madison, WI United States; 6 Department of Psychological and Brain Sciences University of Iowa Iowa City, IA United States

**Keywords:** acceptance and commitment therapy, bipolar disorder, mobile applications, randomized controlled trials, micro-randomized trial, precision medicine, mindfulness

## Abstract

**Background:**

Mobile interventions promise to fill in gaps in care with their broad reach and flexible delivery.

**Objective:**

Our goal was to investigate delivery of a mobile version of acceptance and commitment therapy (ACT) for individuals with bipolar disorder (BP).

**Methods:**

Individuals with BP (n=30) participated in a 6-week microrandomized trial. Twice daily, participants logged symptoms in the app and were repeatedly randomized (or not) to receive an ACT intervention. Self-reported behavior and mood were measured as the energy devoted to moving toward valued domains or away from difficult emotions and with depressive d and manic m scores from the digital survey of mood in BP survey (digiBP).

**Results:**

Participants completed an average of 66% of in-app assessments. Interventions did not significantly impact the average toward energy or away energy but did significantly increase the average manic score m (*P*=.008) and depressive score d (*P*=.02). This was driven by increased fidgeting and irritability and interventions focused on increasing awareness of internal experiences.

**Conclusions:**

The findings of the study do not support a larger study on the mobile ACT in BP but have significant implications for future studies seeking mobile therapy for individuals with BP.

**Trial Registration:**

ClinicalTrials.gov NCT04098497; https://clinicaltrials.gov/ct2/show/NCT04098497

## Introduction

Only 43% of Americans with a mental illness received treatment in 2017, and even fewer among non-Hispanic Black (31%) and Hispanic (33%) individuals [1]. These numbers are partly explained by limited access to therapy, an issue worse for minority groups [2]. Limited access arises due to cost [3], distance [4], and waiting times [5]. Mobile versions of therapy are a promising solution, as they can deliver care at low costs to most people on a schedule that works for them and when they need it the most. In this study, we investigate a mobile version of acceptance and commitment therapy (ACT) for individuals with bipolar disorder (BP).

A mobile version of ACT has many benefits. First, ACT was intended to be effective in general, rather than for specific diagnoses. Its success is attributed to improving a cognitive-behavioral process known as psychological flexibility, which is the ability to pursue things that matter *independent* of negative thoughts and emotions. To emphasize, the goal is not to get rid of unwanted thoughts and emotions, but rather pursue one’s values despite them. Psychological flexibility is theorized to require *awareness* of emotions, thoughts, and behaviors; *openness* to negative thoughts and emotions; and *engagement* with values. Second, ACT is effective in low-dose settings. For example, an online-guided ACT improved depressive symptoms in college students [6], and a mobile ACT improved psychological flexibility [7], suggesting improvements can be achieved with mobile technology [8-11]. Third, ACT teaches specific skills, such as mindfulness, that can be used outside a clinic. Thus, a mobile version of ACT could potentially fill gaps in care for people who would benefit from increased psychological flexibility.

Establishing mobile ACT requires confronting several issues. It is unclear *when* mobile ACT could improve a person’s mood and behavior. One could argue that mobile ACT is more effective when a person is struggling with unwanted negative thoughts and emotions, as this is when psychological flexibility is most warranted. However, mobile ACT may be less effective at these times, given that psychological flexibility is difficult to enact at any moment, let alone when a person is struggling. It is also unclear *who* would benefit the most from mobile ACT. Here again, one could make opposing arguments: mobile ACT is more effective for a person severely impaired by negative thoughts and mood, given that psychological flexibility is likely more important for these individuals, or less effective, given that psychological flexibility is difficult to achieve especially for those impaired. Finally, it is unclear *which* of the 3 subprocesses (ie, awareness, openness, and engagement) would be more effective target. It is important to clarify who should be delivered which ACT intervention and when.

A microrandomized trial (MRT) is a special type of randomized control trial (RCT) for learning to personalize delivery of mobile interventions according to momentary information [12,13]. In a traditional RCT, individuals are randomized once to an intervention condition. Researchers then evaluate the intervention effect on long-term outcomes. An MRT, by contrast, repeatedly randomizes individuals to intervention conditions. Researchers then evaluate the intervention effect on short-term outcomes. Moreover, because interventions are delivered in diverse settings, researchers can also learn how the intervention effect changes according to momentary information.

We conducted 6-week pilot MRTs in 2 populations susceptible to different levels of impairment: individuals with BP and distressed first-generation college students [14]. This paper focuses on the BP group. The overarching goal is to establish mobile ACT as an effective and personalized option for individuals with BP. Primary goals of the present study were safety and feasibility. Secondary goals were effectiveness and personalization. For safety, we examined changes in depressive and manic symptoms over the study. For feasibility, we investigated how often people logged symptoms in the app. For effectiveness and personalization, we examined if delivery of ACT interventions had a short-term effect on mood and behavior and whether this effect changed depending on the person, current mood, and the type of intervention. Results have significant implications for future studies seeking to deliver and personalize mobile therapy for individuals with BP.

## Methods

### Overview

Protocols for this study and the parallel study on distressed first-generation college students were previously published [14]. Briefly, individuals with BP (n=30) participated in a 6-week MRT. Participants were randomized to either receive an ACT intervention or not up to twice each day.

### Ethics Approval

This study was approved by the Institutional Review Boards at the University of Michigan (HUM126732) and the University of Wisconsin (2017-1322) and is registered at clinicaltrials.gov (NCT04098497). The authors assert that all procedures contributing to this work comply with the ethical standards of the relevant national and institutional committees on human experimentation and with the Helsinki Declaration of 1975, as revised in 2008.

### Participants

Individuals with BP were recruited from the Prechter Longitudinal Study of Bipolar Disorder by a research technician [15]. Inclusion criteria were a diagnosis of bipolar disorder (type I, II, not otherwise specified), agreement to be contacted for future research, and access to a smartphone. Each participant received their diagnosis based on the Diagnostic Interview for Genetic Studies [16]. Participants gave their consent over the phone and the consent document was electronically signed. Details on how a target sample size of 30 was determined are found in the published protocol [14].

### Study Components

#### Setup

Participants were mailed an activity tracker (Fitbit Alta HR) and were asked to download a mobile app called Lorevimo (Log, Review, and Visualize your Mood). Lorevimo was designed by the team and is available at Apple App and Google Play stores. Upon first opening the app, participants set typical wake-up and bed times, defining windows for logging symptoms. A morning window was defined as 2-7 hours after the typical wake time. An evening window was defined as 3 hours before and 2 hours after the typical bedtime. Next, they watched a 20-minute video that introduces the ACT Matrix, depicting ACT concepts in four quadrants: identifying and sorting values, internal experiences, avoidance behaviors, and values-based behaviors [17].

#### Initial and Exit Phone Interview

At start and end of the study, mood and health were assessed over the phone by a trained interviewer. Assessments included the Young Mania Rating Scale (YMRS) [18], the Structured Interview Guide for the Hamilton Depression Rating Scale (HRSD) [19], and the 36-Item Short Form Survey [20]. Although self-administration may be more common for the 36-Item Short Form Survey, telephone administration is also considered a valid mode of delivery [21-24].

#### Activity Tracking

Participants wore the Fitbit except during a shower or when charging the Fitbit. The Fitbit tracked sleep, activity, and heart rate.

#### In-App Assessment

Participants logged mood and behavior in the app in the morning and evening. Push notifications were sent as reminders at 2-hour intervals. Mood was self-reported using the 6-item digital survey for mood in bipolar disorder (digiPB) [25,26]. This survey has 3 items (depressed mood, fatigue, and fidgeting) measuring depressive symptoms, 2 items (increased energy and rapid speech) measuring manic symptoms, and 1 item (irritability) measuring both types of symptoms. Each item is rated on a 0-3 ordinal scale. Two scores, *d* and *m*, are computed to measure the severity of depressive and manic symptoms. Participants also answered a 4-item ACT activity survey about current behavior, which are as follows: (1) “In a few words, what behavior are you engaged in right now?” (2) “Does this behavior move you toward who or what matters or away from internal experiences?” (3) “Since [lunchtime or waking up], how much energy was consumed by trying to get rid of unwanted feelings, thoughts, and other internal experiences?” and (4) “Since around lunchtime [lunchtime or waking up], how much energy was consumed by pursuing your values?” The first question was open-ended, the second was binary, and the third and fourth questions were rated on a 0 to 6 ordinal scale.

#### Intervention Delivery

Participants were repeatedly randomized to receive an intervention or not. Participants were *available* for randomization every time they completed an in-app assessment. Each time a participant was randomized, they had 50-50 chance of receiving an intervention. Over the study, a participant could complete up to 84 in-app assessments (=2 per day × 42 days), which means a participant could have been randomized up to 84 different times. When a participant was randomized to *not* receive an intervention, they were navigated back to the home page. When they were randomized to receive an intervention, the participant was navigated to an intervention prompt. The intervention was selected at random from one of the 84 prompts so that each prompt was equal regardless of whether the prompt had previously been delivered or not.

A total of 84 intervention prompts were designed by the team to build ACT skills, organized into 3 ACT concepts (openness, awareness, and engagement) with 28 questions per concept. Openness questions encouraged participants to accept internal experiences rather than engage in avoidance to suppress such experiences. Awareness questions encouraged participants to pay attention to internal experiences and external context and to be present in the moment. Engagement questions encouraged participants to consider their values and the people, things, and qualities that are important to them. These questions also encouraged participants to examine alignment between values and current behavior.

### Outcomes

#### Primary (Feasibility and Safety)

Feasibility was evaluated based on completion of in-app assessments. Safety was evaluated based on changes in YMRS and HRSD scores from baseline to exit, providing low-level evidence (ie, not causal evidence) that the study impacted mood symptoms.

#### Secondary and Exploratory (Effectiveness)

Effectiveness was evaluated based on the effect of intervention delivery on toward and away energy, as measured by the ACT activity survey. We also evaluated intervention effects on the *m* and *d* from the digiBP survey. We also explored intervention effects on individual symptoms and moderation by intervention type, age, sex, diagnosis, and current depressive and manic symptoms prior to randomization.

### Statistical Analysis

For feasibility, we used a 1-sample *z*-test to assess whether participants responded to over half of the assessments per day for over 60% of the days of the intervention period on average. For safety, we used a 1-sample *z*-test to assess whether a mean change in YMRS or HRSD scores was significantly different from zero and a sign test was used to assess whether an equal proportion of individuals saw an increase in YMRS scores as a decrease from baseline to study exit and similarly for HRSD scores.

For effectiveness, we used a weighted and centered least squares method [27,28] to estimate the average effect of delivering an intervention on primary outcomes (toward and away energy) and secondary outcomes (*d* and *m* scores) as a function of time in the study conditional on the participant being available for randomization. A linear working model was used to estimate these effects. A similar approach was used to estimate moderation of intervention effects except for making requisite changes to the linear working model. The study was not powered for moderation analyses; any subsequent findings may be spurious and are, therefore, reported in Multimedia Appendix 1A. Robust SEs were calculated using a sandwich estimator [29]. 

The only source of missingness was if a participant did not complete an in-app assessment at their next assessment window after being randomized. As specified in our protocol [14], additional variables were controlled for that predicted missingness if more than 10% of the data was missing. Potential variables included age, sex, diagnosis, time of day, day in the study, count of prior interventions delivered, count of prior completed in-app assessments, count of prior missing data points, toward energy reported immediately before randomization, and away energy reported immediately before randomization. Linear models were built for the logit function of expected missingness as a function of these potential variables. The best model was selected according to quasi information criterion [30]. Variables in the best model were then controlled for when estimating intervention effects.

For analyses, hypothesis tests were 2-tailed, and the significance threshold was *P*<.05.

## Results

### Participant Flow

Thirty individuals with BP were enrolled between September 2019 and September 2020 (see Multimedia Appendix 1B for the Consolidated Standards Of Reporting Trials diagram). The study ended in October 2020 because enrollment goals were met and data collection was completed. All participants completed the first interview. One participant missed the exit interview and was not included when analyzing safety outcomes. One participant never set up the study app, and 3 participants never linked their Fitbit to the study. Four participants never logged their symptoms and were never randomized to receive an intervention. These 4 participants were not included when analyzing effectiveness outcomes. Six participants never used Fitbit. All participants were included when analyzing feasibility outcomes.

### Sample Characteristics

Table 1 summarizes sample characteristics. They had an average (SD) age of 42.70 (11.11) years and were 60% female. The majority were White (83%), non-Hispanic (93%), and diagnosed with bipolar I disorder (80%).

**Table 1:** Characteristics of the sample population (N=30).

Variables		Value
Age (years), mean (SD)		42.70 (11.11)
Female, n (%)		18 (60)
**Race, n (%)**		
	White	25 (83)
	Black or African American	2 (7)
	Asian	0 (0)
	American Indian or Alaskan Native	1 (3)
	More than 1	2 (7)
	Hispanic	2 (7)
**Diagnosis, n (%)**		
	Bipolar I disorder	24 (80)
	Bipolar II disorder	6 (20)
	Bipolar disorder not otherwise specified	0 (0)
Baseline HRSD^a^ score, mean (SD)		6.20 (5.78)
Baseline YMRS^b^ score, mean (SD)		1.83 (3.29)

^a^HRSD: Hamilton Rating Scale for Depression.

^b^YMRS: Young Mania Rating Scale.

### Safety Outcomes

**Figure 1:**
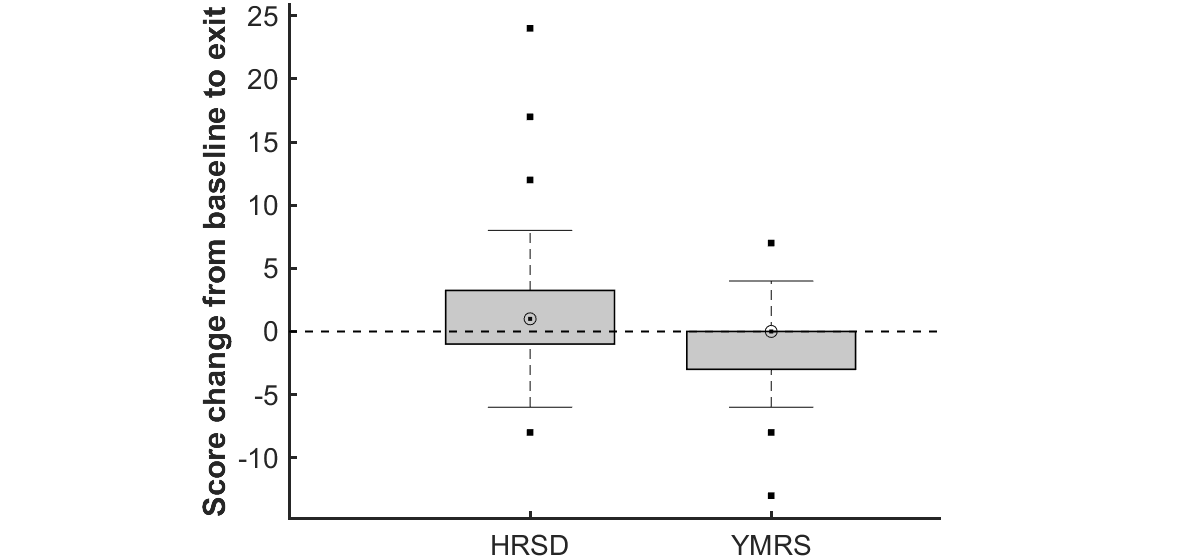
Box plot of change in manic and depressive severity over course of 6-week study, as measured by the HRSD and the YMRS. Extreme values, marked with a dot, are any data points less than the 25th percentile minus 1.5 times the interquartile range or greater than the 75th percentile plus 1.5 times the interquartile range. HRSD: Hamilton Rating Scale for Depression; YMRS: Young Mania Rating Scale.

Figure 1 illustrates the change from baseline to study exit in HRSD and YMRS scores for the 29 participants who completed baseline and exit interviews. Depressive severity increased slightly with an average increase in HRSD score of 2.1 points (*t*_28_=1.75, *P*=.09) and with 15 participants seeing an increase in HRSD scores compared to 9 participants seeing a decrease (mean decrease 63%, *z*=1.22, *P*=.22). Five participants saw no change in HRSD scores. Manic severity decreased slightly with an average decrease in YMRS score of 1.2 points (*t*_28_=1.74, *P*=.09) and with 4 participants seeing an increase in YMRS scores compared to 9 participants seeing a decrease (mean decrease 31%, *z*=−1.39, *P*=.17). Sixteen participants saw no change in YMRS scores.

Given that participation was associated with a slight increase in depressive symptoms, we investigated whether ACT interventions contributed to these changes. Among participants who were randomized at least once, the average HRSD score still increased over the study, but only by 1.2 points (*t*_24_=1.15, *P*=.26) rather than 2.1 points. Average YMRS score decreased by the same 1.2 points (*t*_24_=1.52, *P*=.14) as in the full sample.

### Feasibility

Our second analysis investigated whether participants were available for randomization, that is, whether a participant logged their symptoms in the app at one of the 84 times points. Participants were available for randomization for an average of 66% of the time points. Excluding the 4 participants who never logged symptoms, availability increased to an average of 76% of the time points. In addition, participants were available at least once a day for an average of 74% of the days, which was significantly larger than our prespecified target of 60% (*t*_29_=2.15, *P*=.04). Again excluding participants who never logged symptoms, participants were available at least once a day for an average of 85% of the days.

### Effectiveness

Our final analysis explored whether ACT interventions had a short-term impact on mood and behavior. Primary outcomes were missing for 12% of the times that a participant was randomized. Because this amount was larger than a prespecified cutoff of 10%, all models in this section controlled for the following covariates predicting missingness: time of day, count of prior missing values, count of prior logs, and self-reported toward behavior immediately prior to randomization (see Multimedia Appendix 1C for details on model selection and final models).

Adjusting for these covariates, we found that ACT interventions did not have a significant impact on toward behavior (β=−.006; *z*=−0.11; *P*=.91) or away behavior (β=.093; *z*=1.65; *P*=.10). ACT interventions did, however, significantly increase average depressive score *d* (β=.57, *z*=2.39, *P*=.02) and manic score *m* (β=.19; *z*=2.67; *P*=.008). The day in the study, ranging from day 1 through day 42, did not significantly moderate the effect of the intervention on any of the 4 outcomes.

Given that the ACT interventions worsened mood, we explored the symptoms that might be more greatly impaired and interventions that might be more impairing. Table 2 reports the average effects for all 6 symptoms from the digiBP survey. Symptoms that were significantly impacted by the interventions were fidgeting (β=.130; *z*=2.61; *P*=.009) and irritability (β=.129; *z*=3.34; *P*=.001).

We looked at the type of intervention in 2 ways: the 3 ACT processes (ie, openness, engagement, and awareness) or the 4 quadrants in the ACT matrix (ie, toward behavior, away behavior, internal experiences, and who/what matters). Table 3 reports average effects by intervention type. In the former method, awareness interventions led to a significant increase in average depressive score *d* (β=.65; *z*=2.04; *P*=.04) and manic score (β=.31; *z*=3.04; *P*=.002). Openness or engagement intervention did not lead to a significant increase in any score*.* In the latter method, interventions focused on who or what matters significantly increased average depressive score *d* (β=0.86; *z*=2.54; *P*=.01), whereas interventions focused on away behaviors significantly increased average manic score *m* (β=.92; *z*=2.09; *P*=.04).

**Table 2:** Average effects of acceptance and commitment therapy interventions on individual symptoms.

Outcome	β	95% CI	*z* score	*P* value
Depressed mood	.056	(−.012 to .123)	1.62	.11
Fatigue	.026	(−.081 to .132)	0.47	.13
Fidgeting	.130	(.033 to .227)	2.61	.009
Increased energy	.004	(−.050 to .058)	0.13	.89
Rapid speech	.030	(−.010 to .070)	1.47	.14
Irritability	.129	(.053 to .204)	3.34	.001

**Table 3:** Average effects of interventions on secondary outcomes by intervention type.

Type	Depressive score *d*					Manic score *m*			
	β	95% CI	*z* score	*P* value	β		95% CI	*z* score	*P* value
Engagement	.58	(−.05 to 1.28)	1.82	.07	.19		(−.06 to .43)	1.49	.14
Awareness	.65	(.025 to 1.28)	2.04	.04	.31		(.11 to .50)	3.04	.002
Openness	.45	(−.26 to 1.16)	1.25	.21	.09		(−.24 to .42)	0.54	.59
Toward behaviors	.32	(−.31 to .96)	0.99	.32	.14		(−.09 to .37)	1.22	.22
Away behaviors	.83	(−.63 to 2.29)	1.12	.26	.92		(.06 to 1.79)	2.09	.04
Internal experiences	.48	(−.19 to 1.15)	1.40	.16	.23		(−.06 to .52)	1.53	.13
Who/what matters	.86	(.19 to 1.52)	2.54	.01	.08		(−.17 to .34)	0.65	.52

## Discussion

We presented results from a pilot MRT on delivering mobile ACT interventions to individuals with BP (n=30). This MRT was one of the two parallel studies, with the other focusing on distressed first-generation college students. The goal was to collect evidence to make a go or no-go decision about pursuing a larger study on effectiveness and personalization of mobile ACT in BP. For reasons described below, we concluded such a study is not warranted. Despite this negative conclusion, we discuss several important findings that inform future mobile studies in BP.

A main concern with mobile interventions is whether users sufficiently use the app to have a measurable effect. Thus, we wanted to know if participants met our benchmark for logging symptoms regularly and subsequently being available to receive interventions. The key number here is that participants were available for randomization at an average of 66% of twice-daily time points, because this number determines suitable sample sizes for MRTs [12,31]. Note that participants were not renumerated for interacting with the app. In fact, 66% number includes 4 participants who never logged symptoms. Participants were reminded by push notifications to log symptoms, which is likely to be crucial for achieving high utilization of the app.

Although limited use of the app does not appear to be a limiting factor, safety and effectiveness does appear to be a factor. The first hint that the mobile ACT may have a detrimental effect arose when analyzing safety outcomes. There was a slight 2-point increase in depressive symptoms as measured by the HRSD. Although this increase was nonsignificant, the small sample limits our ability to detect significant changes. On a positive note, there was only a 1-point increase in the HRSD when excluding patients who never were randomized and even a slight 1-point decrease in manic symptoms as measured by the YMRS. Of course, we did not randomize individuals to participate in this study vs not, so we cannot conclude that these changes were due to study participation.

When we look at effectiveness, the picture becomes clearer. Delivering an ACT intervention led to a significant increase in depressive and manic symptoms at the next time period. That is, if a person was delivered an intervention in the morning, they were expected to report higher mood symptoms in the evening than a person who was not delivered an intervention. Similarly, if a person was delivered an intervention in the evening, they were expected to report higher mood symptoms in the morning on the next day than a person who was not delivered an intervention. We looked more closely to determine which symptoms might be more greatly affected. Irritability and fidgeting were greatly affected compared to fatigue, depressed mood, increased energy, or rapid speech. Thus, symptoms induced by ACT interventions were more akin to dysphoric or anxious depression as opposed to anhedonic depression or euphoric mania.

So why might ACT interventions make someone more irritable or agitated? We looked at different factors. Interventions focused on raising a person’s awareness of internal experiences (eg, emotions) had the most significant effect. Furthermore, if a person was currently depressed or manic, then interventions had even larger effect on depressive symptoms. This may be a manifestation of a feature of BP known as emotional reactivity, whereby a person’s emotions react more intensely when provoked [32]. These findings suggest that awareness of negative emotions and thoughts, especially when a person is already experiencing intense mood symptoms, can increase irritability and agitation in individuals with BP.

The downside of raising awareness helps shed light on other digital interventions in BP. A qualitative study from a prior RCT identified common explanations for why individuals with BP did not complete web-based psychoeducation that included difficulties with acute phases of BP and not wanting to think about one’s illness. Moreover, the MONARCA I and II trials found that their mobile intervention group involving computer-based monitoring experienced a nonsignificant increase in average depressive symptoms and nonsignificant decrease in manic symptoms as measured by the HRSD and YMRS [33,34]. The RADMIS trial found their mobile intervention group experienced a nonsignificant decrease in average HRSD scores and a significant increase in average YMRS scores [35].

These findings provide 3 lessons for future mobile studies in BP. The first is that given raising awareness of mood can worsen mood, monitoring mood may carry risks. Put differently, focusing on what is not going well, such as by asking about current mood or behavior, may be distressing for people with BP. Partly in conflict, individuals with BP had previously endorsed raising self-awareness as the best reason for digital self-monitoring of symptoms [36]. Moreover, the World Health Organization recommends that individuals with BP should monitor their mood [37]. One possible compromise would be passive solutions for monitoring mood [38], such as voice patterns [33,34,39], phone keystroke data [40], global positioning system [41], or phone metadata [42]. It is also possible that monitoring of mood *alone* in combination with therapist support may serve a helpful function.

The second lesson is that mood might not be a suitable outcome. As noted earlier, ACT is designed to increase psychological flexibility, that is, not get rid of unwanted thoughts or emotions, but rather pursue what matters despite them. To this point, our primary outcomes were energy devoted to behaviors that move a person toward who or what matters to them, and away from unwanted emotions and thoughts. The only estimated effect of ACT interventions that was near zero was toward energy. Future studies may want to align outcomes with the cognitive behavioral process that is targeted. Given that instability of mood can be as debilitating in BP as mood itself [43,44], a better outcome may be mood stability. Consistency in values-based behavior and workable responses to mood changes may also be an outcome congruent with the goal of psychological flexibility.

The suitability of mood as an outcome may be especially salient when the intervention (like ACT) incorporates mindfulness. Part of mindfulness is increased awareness of symptoms. Through this lens, it is possible that ACT interventions had the desired effect: participants become more aware of unwanted emotions, and consequently, were reporting higher symptoms. This awareness may have temporarily intensified the experience of the emotion but could promote long-term self-efficacy, mindfulness, or psychological flexibility. Furthermore, becoming aware of unpleasant emotions can be both irritating and agitating. Awareness alone may be insufficient to help individuals. Although interventions in openness and engagement were included, interventions were randomized, and thus were not delivered in a particular order that may have benefited those developing awareness of intense and unpleasant emotions. Future work may examine the impact of the order of interventions, such as building skills in awareness, followed by openness, and in parallel, altering behavioral engagement. Fittingly, although the interventions led to higher reported symptoms on average, it did not deter participants from engaging with the app. In fact, we found, it increased the likelihood of logging symptoms at the next time point.

The third lesson is that there might be a better mobile intervention for BP. ACT was chosen for its potential to reach a transdiagnostic audience, with our 2 initial samples chosen at the extreme ends of impairment, but it may be better to focus on interventions specific to BP, such as interpersonal and social rhythms therapy. Alternatively, a mobile intervention may need to be augmented by clinical support. Regardless, it is still notable that a mobile intervention can have a significant effect, though expectedly small (~0.1 standardized effect size), for a pilot study not powered for effectiveness. This information could inform future MRTs on BP as effect sizes are needed, for sample, size calculations [12,31].

There are several limitations to keep in mind. First, we did not collect data on psychological or pharmacological treatment. Thus, we did not control for treatment. Second, our sample was small and relatively homogeneous being 60% female, 83% White, and 93% non-Hispanic. Third, limited data were collected on engagement with the app or with the intervention. The study did not, for example, determine who viewed the introductory ACT video, if individuals would have engaged with the app were to remove the in-app assessments, or if participants engaged with those ACT processes targeted by the intervention. Regarding this last point, however, we did recently publish a preplanned, interim, and qualitative analysis of participant open-ended responses to behavior prompts and intervention prompts [45]. By reading and coding these responses, these qualitative analyses were investigated if participants responded in a way that is congruent with the targeted ACT process and was intended to show intervention fidelity, supporting the study’s reporting of preplanned primary and secondary analyses of the MRT. Fourth, participants wore Fitbit activity trackers, which induce behavioral changes. Fifth, the ACT survey used, including the primary outcomes of toward and away energy, was developed for this study and has yet to be validated.

A final limitation is that the time frame between intervention and assessment, which is roughly half a day, may have been too short to observe behavioral changes induced from ACT. The motivating ACT literature has shown that low-dose versions of ACT have been effective but have not examined how quickly people respond. Therefore, while the present study adds to the ACT literature by demonstrating that ACT interventions can have significant effect on mood in a short time frame, it remains unclear if ACT interventions could similarly affect behavior in this time frame. With limited investigation into a short time frame in the ACT literature, the short time frame for the present study was primarily motivated by the microrandomized trial literature, which has shown that behavioral interventions in other contexts could have effects in time frames as short as 30 minutes [27].

In summary, we presented results from a pilot MRT delivering a mobile ACT intervention in BP. Because the ACT intervention worsened mood, we concluded a larger MRT was not warranted for BP. However, our results are informative for future studies on BP: it is feasible to get measurable effects in MRTs with a small sample size; estimated availability and effects can inform sample sizes for MRTs; self-reported mood may not be the best target; and interventions may need to manage the consequences of raising symptom awareness.

## Appendix

Multimedia Appendix 1 Accompanying appendix reporting (A) Moderation analyses, (B) CONSORT Diagram, (C) Missingness procedure, and (D) Lagged effects.

Multimedia Appendix 2 CONSORT-EHEALTH checklist (V 1.6.1).

## Abbreviations

ACT: acceptance and commitment therapy
BP: bipolar disorder
HRSD: Hamilton Depression Rating Scale
MRT: microrandomized trial
RCT: randomized control trial
YMRS: Young Mania Rating Scale
