# Supplementary material for: Mobile Acceptance and Commitment Therapy in Bipolar Disorder: Microrandomized Trial
Source: JMIR Ment Health. 2023 Apr 20;10:e43164. doi: 10.2196/43164 (PMC10160940; doi:10.2196/43164)
Supplement: Multimedia Appendix 1 [file mental_v10i1e43164_app1.docx]

**Appendix 1**

**A. Moderation analyses**

Table 4 reports estimated interactions between intervention delivery and five potential moderators (age, sex, diagnosis, current *d* score, and current *m* score). Current *m* score was the only significant moderator between intervention delivery and depressive score *d* (*β* = 0.27, *z* = 2.82, *P* = .005). In this case, ACT interventions increased average depressive *d* scores more greatly among individuals currently reporting high manic scores *m*. There were no significant moderators between intervention delivery and manic score *m.*

**Table 4.** Interactions between interventions and covariates on secondary outcomes.

|  | **Depressive score *d*** | | | | **Manic score *m*** | | | |
| --- | --- | --- | --- | --- | --- | --- | --- | --- |
| **Moderator** | **β** | **95% CI** | ***z*** | ***P*** | **β** | **95% CI** | ***z*** | ***P*** |
| Age (years) | -0.03 | (-0.07, 0.02) | -1.23 | .22 | -0.00 | (-0.02, 0.01) | -0.41 | .68 |
| Female | -0.06 | (-1.05, 0.93) | -0.11 | .91 | 0.24 | (-0.03, 0.50) | 1.77 | .08 |
| Bipolar Type II | 0.68 | (-0.12, 1.49) | 1.30 | .20 | -0.05 | (-0.30, 0.20) | -0.40 | .69 |
| Current *d* score | 0.07 | (-0.03, 0.71) | 1.79 | .07 | 0.02 | (-0.02, 0.06) | 0.88 | .38 |
| Current *m* score | 0.27 | (0.08, 0.46) | 2.82 | .005 | 0.03 | (-0.06, 0.11) | 0.56 | .57 |

Since the study was not powered to detect moderation, we highlight moderators trending significant. Biological sex was a marginally significant moderator between intervention delivery and manic score *m* (*β* = 0.24, *z* = 1.77, *P* = .08), whereby ACT interventions increased manic *m* scores more greatly in females. Current depressive *d* score immediately prior to randomization was a marginally significant moderator between intervention delivery and depressive score *d* (*β* = 0.07, *z* = 1.79, *P* = .07), whereby ACT interventions increased depressive *d* scores more greatly among individuals currently reporting high manic scores *d*.

**B. CONSORT Diagram**

**CONSORT 2010 Flow Diagram**

Analysed (n=30)
♦ Excluded from safety analyses (n=1)

♦ Excluded from feasibility analyses (n=0)

♦ Excluded from effectiveness analyses (n=4)

## Analysis

Lost to follow-up (n=1):

♦ Missed exit interview (n=1)

## Follow-Up

Allocated to micro-randomized trial (n=30)

♦ Randomized at least once (n=26)

♦ Were never randomized (n=4)

## Allocation

Enrolled (n=30)

## Enrollment

Excluded (n=80)

♦  Never replied (n=64)

♦  Interested, but study was full (n=16)

Assessed for eligibility (n=110)

**C. Missingness procedure**

The results on effectiveness presented in the main text were recovered by building a series of linear models for mean outcomes. To start, we found that our main effectiveness outcomes (toward and away energy) were missing 12.3% of the time. Recall that a data point is considered *missing* if a person was randomized to an ACT intervention at the current time point but did not submit their self-reported mood or behavior at the next time point. For example, a person may be randomized to receive an ACT intervention in the morning but forget to submit their self-reports in the evening. As a result, they would have a missing nearby outcome for evaluating the effect of being assigned (or not assigned) to receive an ACT intervention.

Since 12.3% is larger than a 10% threshold specified in our published protocol, we decided to control for additional variables in our linear working models. Suitable controls needed to be available prior to randomization and predicted missingness. Candidate variables included time of day (evening vs. morning), count of prior missing outcomes, count of prior submitted assessments, and current toward energy immediately prior to randomization. To identify suitable variables, we built a series of logistic regression models of missingness with the following variables:

1. None
2. Time of day
3. Time of day and count of prior missing outcomes
4. Time of day, count of prior missing outcomes, and count of prior submitted assessments
5. Time of day, count of prior missing outcomes, count of prior submitted assessments, and current toward energy immediately prior to randomization

These models led to progressively smaller values of QICu values, which in the order of the models presented above, were 1237.3, 1203.8, 1124.4, 1083.6, 1081.3. For reference, these models also led to progressively smaller values of QIC: 1252.0, 1220.0, 1138.3, 1094.1, 1089.6. Using a guideline that a 2-point decrease in QICu is a meaningful improvement in model fit, we settled on the final model that included all candidate variables as controls (time of day, count of prior missing outcomes, count of prior submitted assessments, and current toward energy immediately prior to randomization). Thus, these four variables were included as controls in all subsequent models and will be referred henceforth as “controls.”

To provide insight into why a participant might not log symptoms at the next app session after randomization, we report exponentiated coefficients (i.e. adjusted odds ratio) of the best fitting model predicting missingness. Time of day was a significant predictor of missingness, whereby morning in-app sessions were more likely to be missing than evening in-app sessions (aOR = 2.51, *z* = 4.76, *P* < .001). A greater number of prior missing sessions was associated with a significant increase in the likelihood of missingness (aOR = 1.23, *z* = 6.30, *P* < .001), whereas a greater number of prior logging sessions was associated with a significant decrease in the likelihood of missingness (aOR = 1.03, z = 3.71, *P* < .001). Last, current toward energy was associated with a significant increase in the likelihood of missingness (aOR = 1.10, z = 2.01, *P* < .04). In sum, participants were less likely to log in-app sessions if it were morning or if they had missed prior sessions, had not logged many sessions previously, or were reporting higher energy devoted to toward behavior.

Upon settling on what variables to control for, we built linear working models to determine the effect of the intervention on various outcomes. These models are described in **Table C1**. The effects reported in the main text are the coefficients corresponding to the intervention terms in the models. We note that study ‘day’ and its interaction with the intervention were included in the initial models, as was proposed in our published protocol. However, since this interaction was not significant, it was dropped (along with ‘day’) from the remaining models.

**Table C1.** Initial models ran to estimate main effect of intervention delivery on various outcomes.

| Outcome | Independent variables |
| --- | --- |
| Primary: | |
| Toward energy | Intervention, day, day x intervention, controls |
| Away energy | Intervention, day, day x intervention, controls |
| Secondary: | |
| Depressive *d* score | Intervention, day, day x intervention, controls |
| Mania *m* score | Intervention, day, day x intervention, controls |
| Exploratory: | |
| Depressed mood | Intervention, controls |
| Fatigue | Intervention, controls |
| Fidgeting | Intervention, controls |
| Increased energy | Intervention, controls |
| Rapid speech | Intervention, controls |
| Irritability | Intervention, controls |
| Depressive *d* score | Intervention by type (i.e. openness, engagement or awareness), controls |
| Mania *m* score | Intervention by type (i.e. openness, engagement or awareness), controls |
| Depressive *d* score | Intervention by ACT matrix quadrant, controls |
| Mania *m* score | Intervention by ACT matrix quadrant, controls |

We then built linear working models to determine interaction effects between the intervention and various covariates. These models are described in **Table C2**. The effects reported in the main text are the coefficients corresponding to the interaction terms in the models.

**Table C2.** Initial models ran to estimate interaction effects of intervention delivery on various outcomes.

| Outcome | Independent variables |
| --- | --- |
| Depressive *d* score | Intervention, intervention x age, controls |
| Mania *m* score | Intervention, intervention x age, controls |
| Depressive *d* score | Intervention, intervention x sex, controls |
| Mania *m* score | Intervention, intervention x sex, controls |
| Depressive *d* score | Intervention, intervention x diagnosis, controls |
| Mania *m* score | Intervention, intervention x diagnosis, controls |
| Depressive *d* score | Intervention, intervention x current *d* score, controls |
| Mania *m* score | Intervention, intervention x current *d* score, controls |
| Depressive *d* score | Intervention, intervention x current *m* score, controls |
| Mania *m* score | Intervention, intervention x current *m* score, controls |

**D. Lagged effects**

The study design allows us to explore how intervention delivery impacts outcomes, not at the very next in-app session, but the one following that. In other words, we can explore how intervention delivery on one morning can influence mood on the next day’s morning. Such an effect is referred to as a lagged effect. We thus repeated the exact same primary and secondary analyses of effectiveness reported in the main text (e.g., controlled for the same variables), except with the binary variable representing intervention delivery from the prior in-app session replaced by the binary variable representing intervention delivery from two in-app sessions ago. It is important to note that these analyses were not pre-planned. We found that ACT intervention did not have significant lagged effects. That is, intervention delivery from two sessions ago did not have a significant impact on toward behavior (*β* = 0.10, *z* = 1.54, *P* = .12), away behavior (*β* = 0.042; *z* = 0.47, *P* = .64), average depressive score *d* (*β* = 0.11, *z* = 0.49, *P* = .62), or manic score *m* (*β* = 0.033, *z* = 0.35, *P* = .73).
