# Supplementary material for: Mobile Acceptance and Commitment Therapy in Bipolar Disorder: Microrandomized Trial
Source: JMIR Ment Health. 2023 Apr 20;10:e43164. doi: 10.2196/43164 (PMC10160940; doi:10.2196/43164)
Supplement: Multimedia Appendix 2 [file mental_v10i1e43164_app2.pdf]

CONSORT-EHEALTH Checklist V1.6.2 Report	Manuscript Number	43164
**(based on CONSORT-EHEALTH V1.6), available at [http://tinyurl.com/consort-ehealth-v1-6].**		
**Date completed**		
3/18/2023 9:58:33		
**by**		
Amy Cochran		
Mobile Acceptance and Commitment Therapy in Bipolar Disorder: A Micro-Randomized Trial		
TITLE		
**1a-i) Identify the mode of delivery in the title**		
"Mobile Acceptance and Commitment Therapy"		
**1a-ii) Non-web-based components or important co-interventions in title**		
Not relevant, no co-interventions.		
**1a-iii) Primary condition or target group in the title**		
The title includes the phrase: "in Bipolar Disorder"		
ABSTRACT		
**1b-i) Key features/functionalities/components of the intervention and comparator in the METHODS section of the ABSTRACT**		
"Twice daily, participants logged symptoms in-app and were repeatedly randomized to receive an ACT intervention or not."		
**1b-ii) Level of human involvement in the METHODS section of the ABSTRACT**		
"Twice daily, participants logged symptoms in-app and were repeatedly randomized to receive an ACT intervention or not."		
**1b-iii) Open vs. closed, web-based (self-assessment) vs. face-to-face assessments in the METHODS section of the ABSTRACT**		
Closed study with mobile self-assessments and participants recruited offline.		
**1b-iv) RESULTS section in abstract must contain use data**		
"Individuals with BP (n = 30) participated in a 6-week micro-randomized trial." "Participants completed an average 66% of in-app assessments. Interventions did not significantly impact average toward energy or away energy but did significantly increase the average manic score m (P = .008) and depressive score d (P = .02)."		
**1b-v) CONCLUSIONS/DISCUSSION in abstract for negative trials**		
"Findings do not support a larger study on mobile ACT in BP, but have significant implications for future studies seeking mobile therapy for individuals with BP."		
INTRODUCTION		
**2a-i) Problem and the type of system/solution**		
"Mobile versions of therapy are a promising solution, as they can deliver care at low costs to most people on a schedule that works for them and when they need it the most."		
**2a-ii) Scientific background, rationale: What is known about the (type of) system**		
"First, ACT was intended to be effective in general, rather than for specific diagnoses." "Second, ACT is effective in low-dose settings." "Third, ACT teaches specific skills, such as mindfulness, that can be employed outside a clinic."		
**Does your paper address CONSORT subitem 2b?**		
"The overarching goal is to establish mobile ACT as an effective and personalized option for individuals with BP. Primary goals of the present study were safety and feasibility. Secondary goals were effectiveness and personalization."		
METHODS		
**3a) CONSORT: Description of trial design (such as parallel, factorial) including allocation ratio**		
"Briefly, individuals with BP (n= 30) participated in a 6-week MRT." "Each time a participant was randomized, they had 50-50 chance of receiving a intervention."		
**3b) CONSORT: Important changes to methods after trial commencement (such as eligibility criteria), with reasons**		
Not relevant. There were no changes to methods after trial commencement.		
**3b-i) Bug fixes, Downtimes, Content Changes**		
Not relevant. There were no major bugs, downtimes, or content changes over the course of the study.		
**4a) CONSORT: Eligibility criteria for participants**		
"Inclusion criteria was a diagnosis of bipolar disorder (type I, II, not otherwise specified); agreement to be contacted for future research; and access to a smartphone. Each participant received their diagnosis based on the Diagnostic Interview for Genetic Studies [16]."		
**4a-i) Computer / Internet literacy**		
Participants were recruited from a larger cohort that tends to have high computer literacy. In addition, consent involved a electronic signature. Thus, it was assumed participants had computer / internet literacy.		
**4a-ii) Open vs. closed, web-based vs. face-to-face assessments:**		
"Individuals with BP were recruited from the Prechter Longitudinal Study of Bipolar Disorder by a research technician [15]." "At study start and end, mood and health were assessed over the phone by a trained interviewer." "In-app assessment. Participants logged mood and behavior in-app in the morning and evening."		
**4a-iii) Information giving during recruitment**		
"Participants were consented over the phone and electronically signed the consent document."		
**4b) CONSORT: Settings and locations where the data were collected**		
Not relevant; all data was collected over the phone or in the app.		
**4b-i) Report if outcomes were (self-)assessed through online questionnaires**		
"Mood was self-reported using the 6-item digital survey for mood in bipolar disorder (digiPB) [25,26]" "Participants also answered a 4-item ACT activity survey about current behavior:"		
**4b-ii) Report how institutional affiliations are displayed**		
All participants were recruited from a larger cohort. Any potential bias due to displayed affiliations may already be present in the larger cohort study.		
**5) CONSORT: Describe the interventions for each group with sufficient details to allow replication, including how and when they were actually administered**		
**5-i) Mention names, credential, affiliations of the developers, sponsors, and owners**		
Study app is not proprietary. The authors developed the app.		
**5-ii) Describe the history/development process**		
We do not discuss the history/development process of the study app in the present paper, but point the reader to the protocol paper, where some of this information is provided. Regarding evaluation, we mention two prior papers.		
**5-iii) Revisions and updating**		
The content app/intervention was frozen during the trial. There are no dynamic components, except the following: "The intervention was selected at random from one of the 84 prompts."		
**5-iv) Quality assurance methods**		
The mobile interventions were developed by one of the authors, who has expertise in in-person version of the intervention. In addition, the paper cites previously published work exploring intervention fidelity.		
**5-v) Ensure replicability by publishing the source code, and/or providing screenshots/screen-capture video, and/or providing flowcharts of the algorithms used**		
Screenshots and details on the app/intervention can be found in the published protocol.		
**5-vi) Digital preservation**		
https://apkpure.com/lorevimo/io.appery.project488912 (archived version)		
**5-vii) Access**		
Access to the app and Fitbit was free to all participants. The app was downloaded from Apple App and Google Play stores.		
**5-viii) Mode of delivery, features/functionalities/components of the intervention and comparator, and the theoretical framework**		
The study app and intervention are detailed in the published protocol, which the paper cites.		
**5-ix) Describe use parameters**		
"participants set typical wake and bed times, defining windows for logging symptoms."		
**5-x) Clarify the level of human involvement**		
Human involvement was limited to phone calls during informed consent and during initial and exit interventions and emails to troubleshoot.		
**5-xi) Report any prompts/reminders used**		
"Push notifications were sent as reminders at 2-hour intervals."		
**5-xii) Describe any co-interventions (incl. training/support)**		
Not relevant, there were no co-interventions.		

**6a) CONSORT: Completely defined pre-specified primary and secondary outcome measures, including how and when they were assessed**		
"Primary (feasibility and safety). Feasibility was evaluated based on completion of in-app assessments. Safety was evaluated based on changes in YMRS and SIGH-D scores from baseline to exit" "Secondary and exploratory (effectiveness). Effectiveness was evaluated based on the effect of intervention delivery on toward and away energy, as measured by the ACT Activity Survey."		
**6a-i) Online questionnaires: describe if they were validated for online use and apply CHERRIES items to describe how the questionnaires were designed/deployed**		
Our secondary outcomes were from digiBP, which is designed and validated for digital use. Survey for primary outcomes has not been validated.		
**6a-ii) Describe whether and how "use" (including intensity of use/dosage) was defined/measured/monitored**		
"Participants logged mood and behavior in-app in the morning and evening." "Each time a participant was randomized, they had 50-50 chance of receiving a intervention."		
**6a-iii) Describe whether, how, and when qualitative feedback from participants was obtained**		
No qualitative feedback, except for open-ended responses to behavioral and intervention prompts.		
**6b) CONSORT: Any changes to trial outcomes after the trial commenced, with reasons**		
Not relevant; all data was collected over the phone or in the app.		
**7a) CONSORT: How sample size was determined**		
**7a-i) Describe whether and how expected attrition was taken into account when calculating the sample size**		
To account for attrition in power and sample calculations, it was assumed that participants would respond 80% of the time.		
**7b) CONSORT: When applicable, explanation of any interim analyses and stopping guidelines**		
"Primary (feasibility and safety). Feasibility was evaluated based on completion of in-app assessments. Safety was evaluated based on changes in YMRS and SIGH-D scores from baseline to exit" "Secondary and exploratory (effectiveness). Effectiveness was evaluated based on the effect of intervention delivery on toward and away energy, as measured by the ACT Activity Survey."		
**8a) CONSORT: Method used to generate the random allocation sequence**		
Randomization was performed automatically in-app.		
**8b) CONSORT: Type of randomisation; details of any restriction (such as blocking and block size)**		
"Each time a participant was randomized, they had 50-50 chance of receiving a intervention." "The intervention was selected at random from one of the 84 prompts so that each prompt was equally regardless of whether the prompt had previously been delivered or not."		
**9) CONSORT: Mechanism used to implement the random allocation sequence (such as sequentially numbered containers), describing any steps taken to conceal the sequence until interventions were assigned**		
Randomization was performed automatically by the study app.		
**10) CONSORT: Who generated the random allocation sequence, who enrolled participants, and who assigned participants to interventions**		
A research technician enrolled participants. The random allocation sequence and assignment was automatically done by the study app.		
**11a) CONSORT: Blinding - If done, who was blinded after assignment to interventions (for example, participants, care providers, those assessing outcomes) and how**		
**11a-i) Specify who was blinded, and who wasn't**		
Neither participants or researchers were blinded.		
**11a-ii) Discuss e.g., whether participants knew which intervention was the "intervention of interest" and which one was the "comparator"**		
Not relevant, because of micro-randomized trial. Every participant was repeatedly randomized. Participants knew if they received an intervention.		
**11b) CONSORT: If relevant, description of the similarity of interventions**		
Not relevant, because of micro-randomized trial. Every participant was repeatedly randomized to receive or not receive an intervention.		
**12a) CONSORT: Statistical methods used to compare groups for primary and secondary outcomes**		
"For effectiveness, we used a weighted and centered least squares method [27,28] to estimate the average effect of delivering a intervention on primary outcomes (toward and away energy) and secondary outcomes (d and m scores) as a function of time in the study conditional on the participant being available for randomization."		
**12a-i) Imputation techniques to deal with attrition / missing values**		
"As specified in our protocol [14], additional variables were controlled for that predicted missingness if more than 10% of the data was missing." "Linear models were built for the logit function of expected missingness as a function of these potential variables. The best model was selected according to quasi information criterion (QIC) [30]."		
**12b) CONSORT: Methods for additional analyses, such as subgroup analyses and adjusted analyses**		
"We also explored intervention effects on individual symptoms and moderation by intervention type, age, sex, diagnosis, and current depressive and manic symptoms prior to randomization."		
RESULTS		
**13a) CONSORT: For each group, the numbers of participants who were randomly assigned, received intended treatment, and were analysed for the primary outcome**		
"One participant missed the exit interview and were not included when analyzing safety outcomes." "These four participants were not included when analyzing effectiveness outcomes." "All participants were included when analyzing feasibility outcomes."		
**13b) CONSORT: For each group, losses and exclusions after randomisation, together with reasons**		
All individuals randomized were included in effectiveness analyses.		
**13b-i) Attrition diagram**		
We did not include an attrition diagram. We note "Participants were available for randomization for an average of 66% of the time points."		
**14a) CONSORT: Dates defining the periods of recruitment and follow-up**		
"Thirty individuals with BP were enrolled between September 2019 and September 2020 (see Appendix B for CONSORT diagram). The study ended in October 2020, since enrollment goals were met and data collection was completed."		
**14a-i) Indicate if critical "secular events" fell into the study period**		
Not relevant, no critical secular events.		
**14b) CONSORT: Why the trial ended or was stopped (early)**		
"The study ended in October 2020, since enrollment goals were met and data collection was completed."		
**15) CONSORT: A table showing baseline demographic and clinical characteristics for each group**		
Provided in Table 1 of the paper.		
**15-i) Report demographics associated with digital divide issues**		
"They had an average (SD) age of 42.70 (11.11) years and were 60% female. The majority were White (83%), non-Hispanic (93%), and diagnosed with Bipolar I (80%)."		
**16a) CONSORT: For each group, number of participants (denominator) included in each analysis and whether the analysis was by original assigned groups**		
**16-i) Report multiple "denominators" and provide definitions**		
"One participant missed the exit interview and were not included when analyzing safety outcomes" "These four participants were not included when analyzing effectiveness outcomes" "All participants were included when analyzing feasibility outcomes."		
**16-ii) Primary analysis should be intent-to-treat**		
Not relevant, because of micro-randomized trial design. Only users of the app are randomized.		
**17a) CONSORT: For each primary and secondary outcome, results for each group, and the estimated effect size and its precision (such as 95% confidence interval)**		
"ACT interventions did not have a significant impact on toward behavior ($\beta = -0.006$, $z = -0.11$, $P = .91$) or away behavior ($\beta = 0.093$; $z = 1.65$, $P = .10$). ACT interventions did, however, significantly increase average depressive score d ($\beta = 0.57$, $z = 2.39$, $P = .02$) and manic score m ($\beta = 0.19$, $z = 2.67$, $P = .008$)."		
**17a-i) Presentation of process outcomes such as metrics of use and intensity of use**		
"Participants were available for randomization for an average of 66% of the time points."		
**17b) CONSORT: For binary outcomes, presentation of both absolute and relative effect sizes is recommended**		
Not relevant, no effect sizes for binary outcomes.		
**18) CONSORT: Results of any other analyses performed, including subgroup analyses and adjusted analyses, distinguishing pre-specified from exploratory**		
"The study was not powered for moderation analyses; any subsequent findings may be spurious and are therefore reported in Appendix A."		
**18-i) Subgroup analysis of comparing only users**		
We present subgroup safety analyses for those individuals who specifically were randomized to intervention at least once. Because of the micro-randomized trial design, bias arises from a source other than non-use of the app.		
**19) CONSORT: All important harms or unintended effects in each group**		
"Depressive severity increased slightly with an average increase in HRSD score of 2.1 points ($t = 1.75$, $df = 28$, $P = .09$)" "Manic severity decreased slightly with an average decrease in YMRS score of 1.2 points ($t = 1.74$, $df = 28$, $P = .09$)."		
**19-i) Include privacy breaches, technical problems**		
There were no privacy breaches or technical problems during the study.		
**19-ii) Include qualitative feedback from participants or observations from staff/researchers**		
The paper does not include any qualitative feedback, except for open-ended responses that participants provided for behavioral and intervention prompts.		

DISCUSSION		
**20) CONSORT: Trial limitations, addressing sources of potential bias, imprecision, multiplicity of analyses**		
**20-i) Typical limitations in ehealth trials**		
Limitations are noted in the Discussion.		
**21) CONSORT: Generalisability (external validity, applicability) of the trial findings**		
**21-i) Generalizability to other populations**		
"Second, our sample was small and relatively homogeneous being 60% female, 83% White, and 93% Non-Hispanic."		
**21-ii) Discuss if there were elements in the RCT that would be different in a routine application setting**		
In a routine application setting, it is possible that the intervention would not be repeatedly randomized, but rather delivered on a regular schedule.		
**22) CONSORT: Interpretation consistent with results, balancing benefits and harms, and considering other relevant evidence**		
**22-i) Restate study questions and summarize the answers suggested by the data, starting with primary outcomes and process outcomes (use)**		
"The goal was to collect evidence to make a go or no-go decision about pursuing a larger study on effectiveness and personalization of mobile ACT in BP. For reasons described below, we concluded such a study is not warranted. Despite this negative conclusion, we discuss several important findings that inform future mobile studies in BP."		
**22-ii) Highlight unanswered new questions, suggest future research**		
"our results are informative for future studies on BP: it is feasible to get measurable effects in MRTs with a small sample size; estimated availability and effects can inform sample sizes for MRTs; self-reported mood may not be the best target; and interventions may need to manage consequences of raising symptom awareness."		
Other information		
**23) CONSORT: Registration number and name of trial registry**		
"Clinicaltrials.gov NCT04098497"		
**24) CONSORT: Where the full trial protocol can be accessed, if available**		
"Protocols for this study and the parallel study on distressed first-generation college students were previously published [14]."		
**25) CONSORT: Sources of funding and other support (such as supply of drugs), role of funders**		
Information is provided under Acknowledgements.		
**X26-i) Comment on ethics committee approval**		
"This study was approved by institutional review boards at the University of Michigan (HUM126732) and the University of Wisconsin (2017-1322) and is registered at clinicaltrials.gov (NCT04098497)."		
**x26-ii) Outline informed consent procedures**		
"Participants were consented over the phone and electronically signed the consent document."		
**X26-iii) Safety and security procedures**		
Data collected by app was stored with a coded identifier; link was stored in Redcap.		
**X27-i) State the relation of the study team towards the system being evaluated**		
The authors developed the intervention and the app being used for the present study.		
